# Time Required for Root Canal Retreatment Using Continuous Rotation, Reciprocation, and Optimum Torque Reverse Motions: An In-Vitro Study

**DOI:** 10.7759/cureus.67786

**Published:** 2024-08-26

**Authors:** Tarek Abboud, Mouhammad Al-Tayyan, Hassan Achour, Yasser Alsayed Tolibah

**Affiliations:** 1 Endodontics, Damascus University, Damascus, SYR; 2 Pediatric Dentistry, Damascus University, Damascus, SYR

**Keywords:** reciprocating motion, continuous rotational motion, otr motion, working time, endodontic retreatment

## Abstract

Objective

This study aims to evaluate the time required for canal preparation using three different movement kinematics during retreatment: continuous rotational motion, reciprocating motion, and optimum torque reverse (OTR) motion.

Materials and methods

The sample comprised 45 single-canal mandibular first premolars. The crowns were sectioned to standardize the root length to 16 mm. The root canals were prepared using the AF Gold mechanical preparation system (25/06). The roots were obturated using the lateral condensation technique and kept at 100% humidity at 37°C for seven days. The sample was randomly divided based on the movement pattern used during retreatment into three groups (N = 15): group 1 is continuous rotational motion using the ProTaper Universal Retreatment system; group 2 is reciprocating motion using the WaveOne Gold system; and group 3 is OTR motion using the ProTaper Universal Retreatment system. The retreatment time was measured in seconds by summing two times: T1 (time to reach the apex) and T2 (time to achieve adequate cleaning). The data were statistically analyzed using a one-way ANOVA with a significance level of α = 0.05.

Results

The results showed that the time required for canal preparation during retreatment with WaveOne Gold files using reciprocating motion was significantly longer than the time needed with ProTaper Universal Retreatment files using continuous rotary motion or OTR motion (P < 0.05).

Conclusions

The use of OTR motion did not affect the canal preparation time when used with files designed for continuous rotary motion retreatment. The use of WaveOne Gold files for canal preparation during retreatment was associated with a longer working time than ProTaper Universal Retreatment files.

## Introduction

Endodontic treatment failure is often associated with persistent infection or secondary infection due to inadequate cleaning, shaping, and root canal system obturation. This makes endodontic retreatment the primary treatment option [1]. However, retreatment procedures are a demanding and time-consuming process prone to various procedural errors. Selecting the appropriate case for the retreatment procedure requires careful assessment of the prognosis of the tooth and weighing the advantages and disadvantages of the intervention. Additionally, the time required for retreatment plays a significant role in patient acceptance and specialist comfort during the procedure [2]. Various techniques have been proposed to remove root canal filling materials, including manual files, nickel-titanium rotary instruments, Gates Glidden drills, ultrasonic devices, lasers, and the use of solvents. Traditionally, removing gutta-percha with hand files can be tedious and time-consuming, especially when the root canal filling material is well-condensed [3].

Nickel-titanium (NiTi) alloy development and the subsequent transition to rotary preparation marked the beginning of a new era in endodontics. Specialized rotary systems for retreatment have been developed, reducing the dentist’s working time and facilitating the preparation process, thereby increasing efficiency and safety [4]. Advancements in endodontics have not been limited to the development of file alloys. Different movement patterns have also been introduced. In 2008, Yared proposed a new concept of reciprocating motion [5]. Manufacturers subsequently introduced single-file reciprocating systems, such as WaveOne (Dentsply Maillefer, Ballaigues, Switzerland), a system treated with heat during manufacturing to enhance cyclic fatigue resistance and increase instrument flexibility. Reciprocating files have demonstrated better mechanical behavior compared to continuous rotary systems, showing higher resistance to cyclic fatigue [6]. A new preparation technique has been developed that includes the Elements Motor (Sybron Endo, Orange, United States) and the TF Adaptive® file system by SybronEndo. This unique patented movement adapts automatically to the pressure applied to the instrument within the canal. It combines the advantages of continuous rotary and reciprocating motions, reducing the risk of instrument breakage while maintaining performance [7]. Subsequently, JMorita (Kyoto, Japan) developed the OTR movement to leverage the benefits of reciprocating motion while minimizing its drawbacks. The OTR movement has shown greater resistance to cyclic fatigue in comparison with continuous rotary motion while maintaining optimal cutting efficiency [8]. This in-vitro study was conducted to evaluate the efficacy of continuous rotary motion, reciprocating motion, and OTR motion in removing root canal filling materials by assessing the time required for removal.

## Materials and methods

Study design and settings

This study was a controlled, randomized laboratory investigation, conducted at the Department of Endodontics, Faculty of Dentistry, Damascus University, Damascus, Syria, designed to compare the time required for three types of mechanical file movements during endodontic retreatment. This study received ethical approval from the Local Research Ethics Committee of the Faculty of Dentistry, Damascus University (UDDS-76-20122021/SRC-1428) and was funded by Damascus University (funder no. 501100020595).

Sample size calculation

Based on a previous investigation [9], the sample size for this current study was determined utilizing G* Power 3.1.9.4 (Heinrich-Heine-Universität, Düsseldorf, Germany). In the ANOVA analysis, sample sizes of 15 were derived for each of the three groups, resulting in a total sample of 45 subjects. This configuration yields an effect size (f) of 0.52 (which was calculated according to the change in the time required to remove previous root canal obturation materials using different rotary systems), the maximum, and 85% power to discern disparities at a significance level α = 0.05.

Inclusion and exclusion criteria

The inclusion criteria for this study were as follows: single-rooted closed-apex premolars with roots free of fractures or caries, closed and non-resorbed apices, and either straight roots or roots with a curvature not exceeding 5-10 degrees. Radiographic imaging, performed using an intraoral sensor (Ez Sensor HD, VaTech, Korea), was conducted in both buccolingual and mesiodistal directions to identify any anatomical defects. Subsequently, using ImageJ (Fiji) 2019 software, the curvature was determined to determine the included premolars. On the other hand, if premolars showed root resorptions, calcified canals, fractures, or previous obturations on the periapical radiographs, they were excluded. It is worth noting that in the event of a file separation during the initial preparation or retreatment, or if preparation errors such as ledging occurred, the entire sample was to be excluded and replaced with a new one. However, fortunately, this study did not record any such issues. As a result, forty-five premolars met the criteria and were included in the current study.

Group allocation

The sample was randomly divided into three groups using the randomization website (www.randomization.org). Each group consisted of 15 teeth.

Group 1: Continuous Rotational Motion Using ProTaper Universal Retreatment

Continuous rotary motion using ProTaper Universal Retreatment files (Dentsply Maillefer, Ballaigues, Switzerland) at a rotational speed of 500 rpm, following the manufacturer's instructions.

Group 2: Reciprocating Motion Using WaveOne Gold

Reciprocating motion using WaveOne Gold files (Dentsply Maillefer, Ballaigues, Switzerland) with 180° counterclockwise and 60° clockwise rotational angles.

Group 3: OTR Motion Using ProTaper Universal Retreatment

OTR motion using ProTaper Universal Retreatment files (Dentsply Maillefer, Ballaigues, Switzerland) at a rotational speed of 500 rpm for the rotary part of the motion, with 90° counterclockwise and 180° clockwise rotational angles for the reciprocating part of the motion.

Preparation and storage

After extraction, the teeth were immersed in a 5.25% sodium hypochlorite solution for one minute and then stored in saline until use. Subsequently, a mark was made on each tooth at 16 mm from the apical foramen using a caliper to standardize the tooth lengths, and the teeth were sectioned with a diamond disc mounted on a straight handpiece. An access cavity was prepared using a 1 mm diamond bur. After that, canal patency was confirmed using #10 and #15 K-files.

Initial preparation phase

The working length was determined by subtracting 1 mm from the total length, and Ni-Ti rotary instruments from Fanta were used up to size 25 with a 6% taper to perform the endodontic treatment, following the sequence of files in the AF Gold system (Fanta Dental, Shanghai, China). About 2.5 ml of 5.25% NaOCl was used for irrigating all canals between each instrument change. A final irrigation was performed with 5 ml of 17% EDTA for 30 seconds, followed by rinsing with 5 ml of saline. All canals were then dried using paper points.

Lateral compaction obturation phase

The canals were dried using paper points (Sure-endo, Gyeonggi-do, Korea), and the master cone fit (25/04) was verified. The root canals were then obturated using the lateral compaction technique with a resin-based sealer, ADSeal (Meta Biomed, Chungcheong Buk-do, Korea), and gutta-percha (Sure-endo, Gyeonggi-do, Korea). Radiographs in both mesiodistal and buccolingual directions were taken using an intraoral sensor (Ez Sensor HD) to ensure the quality of the root canal filling. The excess gutta-percha was removed using a heated plugger, and the canal orifice was sealed with a temporary filling material (Cavit-G; 3M ESPE, Seefeld, Germany). The teeth were stored in 100% humidity at 37°C for seven days to ensure the complete setting of the sealer.

Root canal retreatment procedures

About 2 mm of the gutta-percha at the coronal part was removed using a size four Gates Glidden bur (Mani, Utsunomiya, Japan) in the three retreatment groups.

Group 1

ProTaper Universal Retreatment (Dentsply Maillefer, Ballaigues, Switzerland) system was used. It consists of a D1 file (30/0.09) for removing the filling material from the coronal third, a D2 file (25/0.08) for the middle third, and a D3 file (20/0.07) for the apical third.

The Tri Auto ZX2 device (JMorita, Kyoto, Japan) was set to continuous rotational mode at 500 rpm as per the manufacturer's instructions [10]. The crown-down technique was employed until reaching the full working length. Retreatment was considered complete when the D3 file reached the working length without the ability to remove additional filling material or upon instrument separation [10]. Each canal was irrigated with 5 ml of distilled water during retreatment [11].

Group 2

WaveOne Gold Primary file (25/0.07 variable taper) (Dentsply Maillefer, Ballaigues, Switzerland) was used with the VDW silver rotary device (VDW GmbH, Munich, Germany) set to the WaveOneall mode. Files were used in an in-and-out motion no more than four times with minimal apical pressure, followed by cleaning the file and removing debris. Retreatment was considered complete when the file reached the working length without the ability to remove additional filling material or upon instrument separation [10]. Each canal was irrigated with 5 ml of distilled water during retreatment [11].

Group 3

ProTaper Universal Retreatment (Dentsply Maillefer, Ballaigues, Switzerland) system was used. It consists of a D1 file (30/0.09) for removing the filling material from the coronal third, a D2 file (25/0.08) for the middle third, and a D3 file (20/0.07) for the apical third.

The Tri Auto ZX2 device (JMorita, Kyoto, Japan) was set to OTR mode at 500 rpm and 1 Ncm torque as per the manufacturer's instructions. The crown-down technique was employed until reaching the full working length. Retreatment was considered complete when the D3 file reached the working length without the ability to remove additional filling material, or upon instrument separation [10]. Each canal was irrigated with 5 ml of distilled water during retreatment [11]. The irrigation needle (Endo-top, China) was placed as deeply as possible into the canal without binding, but no deeper than the pre-determined working length.

A new file was used for each canal in all groups according to the motion kinematic employed. It is worth mentioning that all initial treatment and retreatment procedures were performed by the same specialist (TA), who had four years of experience in endodontics.

Working time measurement

The working time for retreatment was divided into two parts: T1: the time required to reach the apex, excluding the time spent changing instruments; and T2: the time required to achieve sufficient canal debridement (no visible remnants of filling material on the file) [4]. The total retreatment time was the sum of T1 and T2, measured in seconds, which was recorded using a digital timer (Simex, Persiceto, Italy).

Statistical analysis

The collected data were tabulated and analyzed using SPSS Statistics for Windows, Version 13 (Released 2000; SPSS Inc., Chicago, United States). Shapiro-Wilk test indicated the normal distribution of retreatment time required among the three groups (P > 0.05), so the comparison between groups was performed using the one-way ANOVA and Bonferroni tests. The level of significance was set at α = 0.05.

## Results

The study sample consisted of 45 human mandibular first premolars that were divided into three equal groups based on the type of rotary movement used (continuous rotation, reciprocation, and OTR).

Time required to reach the apex (T1) results

A one-way ANOVA test was conducted to assess the significance of differences in the time required to reach the apex (measured in seconds) among the groups as shown in Table 1.

**Table 1:** Descriptive analyses of the time required to reach the apex (T1) in seconds and the one-way ANOVA test result ^*^: one-way ANOVA test; OTR: optimum torque reverse

Motion kinematics	Tooth number	Mean ± standard deviation	Minimum	Maximum	F-value	^*^P-value
Continuous rotational motion	15	74.00 ± 15.35	53	103	45.084	<0.001
Reciprocating motion	15	516.80 ± 219.06	135	961		
OTR motion	15	110.00 ± 29.63	61	166		

One-way ANOVA test revealed significant differences in the time required to reach the apex (T1) in seconds among groups (P < 0.001). To determine which of the movement patterns differed from the others in terms of the time required to reach the apex (in seconds), pairwise comparisons were conducted using the Bonferroni test as shown in Table 2.

**Table 2:** Bonferroni test between groups results in T1 OTR: optimum torque reverse

Group	Group	Mean difference	P-value
Continuous rotational motion	OTR motion	-36.00	1.000
	Reciprocating motion	-442.80	<0.001
Reciprocating motion	OTR motion	-406.80	<0.001

The study findings suggest that the T1 in the reciprocation motion group was longer compared to the continuous rotational motion group and the OTR motion group individually.

Time required to achieve sufficient canal debridement (T2) results

A one-way ANOVA test was conducted to assess the significance of differences in the time required to achieve sufficient canal debridement (measured in seconds) among the groups as shown in Table 3.

**Table 3:** Descriptive analyses of the time required to achieve sufficient canal debridement (T2) in seconds and the one-way ANOVA test result ^*^: one-way ANOVA test; OTR: optimum torque reverse

Motion kinematics	Tooth number	Mean ± standard deviation	Minimum	Maximum	F-value	^*^P-value
Continuous rotational motion	15	82.13 ± 39.38	34	140	3.110	0.057
Reciprocating motion	15	147.13 ± 103.57	28	360		
OTR motion	15	107.00 ± 41.59	52	171		

The previous table indicates that there are no statistically significant differences in the time to achieve adequate cleaning (in seconds) among the three groups.

Total retreatment time required result

A one-way ANOVA test was conducted to assess the significance of differences in the total retreatment time required (measured in seconds) among the groups as shown in Table 4.

**Table 4:** Descriptive analyses of the total retreatment time required in seconds and the one-way ANOVA test result *: one-way ANOVA test; OTR: optimum torque reverse

Motion kinematics	Tooth number	Mean ± standard deviation	Minimum	Maximum	F-value	^*^P-value
Continuous rotational motion	15	156.13 ± 36.12	115	224	77.786	<0.001
Reciprocating motion	15	663.93 ± 185.58	398	1084		
OTR motion	15	217.00 ± 29.15	179	262		

One-way ANOVA test revealed significant differences in the total retreatment time required in seconds among groups (P < 0.001). To determine which of the movement patterns differed from the others in terms of the total retreatment time required (in seconds), pairwise comparisons were conducted using the Bonferroni test as shown in Table 5.

**Table 5:** Bonferroni test between groups results in the total retreatment time required OTR: optimum torque reverse

Group	Group	Mean difference	P-value
Continuous rotational motion	OTR motion	-60.87	0.696
	Reciprocating motion	-507.80	<0.001
Reciprocating motion	OTR motion	-446.93	<0.001

The study findings suggest that the total retreatment time required in the reciprocation motion group was longer compared to both the continuous rotational motion group and the OTR motion group individually.

## Discussion

Non-surgical retreatment aims to re-establish the conditions necessary for the periapical tissues to heal within a short time frame [12]. Biofilms can form within the root canal obturation material in cases of failed endodontic treatments, and necrotic tissues or bacteria covered by the obturation material can cause pain or periapical inflammation [13]. Therefore, during retreatment, it is essential to remove the root canal obturation materials to reduce the number of microorganisms safely, effectively, and quickly to achieve treatment success, patient satisfaction, and specialist comfort [1,2]. After reaching the apex again, the previous obturation materials are entirely removed, followed by cleaning and shaping of the canal system and then the final obturation [14].

The advantages of using rotary instruments include maintaining the canal shape, reducing working time, and minimizing practitioner fatigue, while the disadvantages include a higher rate of instrument separation [15], apical extrusion of the filling material, and preparation debris [16]. This study focused on one variable: the time required for retreatment as an evaluation method. This study aimed to assess the efficiency of using reciprocating motion with the WaveOne Gold system and continuous rotary motion and OTR motion with the ProTaper Universal Retreatment system in the speed of removing root canal filling materials. The OTR motion, recently introduced, has reduced rotational fatigue without affecting tool efficiency [17].

Research has always been aimed at finding a quick, safe, and effective method for removing root canal-obturation materials. Although single-file reciprocating systems like WaveOne have demonstrated better mechanical behavior compared to continuous rotary systems by showing higher resistance to rotational fatigue [6], conflicting results have been reported regarding the time required to remove root canal obturation materials. De Souza et al. reported that reciprocating motion is faster at removing root canal filling materials compared to continuous rotation [9], while Faus-Matoses et al. indicated that the time required for retreatment was the same when comparing reciprocating motion with continuous rotary motion [18]. To the best of the researcher's knowledge, no prior studies have evaluated the time required to complete the endodontic retreatment using the OTR system. Therefore, the aim of this study was to focus on the time required for endodontic retreatment using a continuous rotation system, the Reciproc system, and the OTR system.

The study sample consisted of mandibular premolars with straight canals to facilitate uniform canal anatomy. The crowns were removed to standardize the working length and approximate amount of root canal filling material in the sample and to exclude the influence of variables such as the tooth's crown anatomy and access cavity design, leading to a more reliable study [19]. Root canal filling was performed using the lateral compaction technique with AD seal, which is the most common technique [20]. The sample was stored at 37°C in 100% relative humidity for seven days to ensure the complete setting of the sealer [21]. Solvents were not used in this study. Although solvents facilitate penetration of root canal filling materials [22], they have been reported to increase retreatment time as they form a slurry-like mixture when interacting with filling materials, adhering to canal walls and being difficult to remove [23]. Additionally, residues of root canal filling materials were found within dentinal tubules when using solvents [24]. Distilled water was used as an irrigant during canal cleaning as it is inert and has no solvent effect. Given that the manufacturer's instructions for the ProTaper Retreatment Universal system indicate that the tool reaching the apical third is D3, with a size of #20, the primary WaveOne Gold file with a size of #25 was chosen as it is the closest reciprocating file size to D3 [19]. In fact, the design of the file blades used varies between different endodontic retreatment systems. Due to differences in blade orientation, systems that allow continuous rotational motion are not compatible with the Reciproc system. However, those compatible with the Reciproc system are also compatible with OTR motion. As a result, standardizing files across all three systems was not applicable. The time required for canal preparation was measured in some studies without excluding irrigation and tool-changing times [25]. Including these times may affect the accuracy of the results, as the total time measured is influenced by more than one variable. Other studies measured the preparation time using the operating time of the preparation device, which may be more accurate than using a stopwatch, as the time measurement is linked to the device's operation switch [10]. This method was not used due to the lack of this feature in the preparation device used.

According to the results of this study, reciprocating motion took longer to perform retreatment with statistically significant differences, especially in the time required to reach the apex. In contrast, there were no statistically significant differences when comparing the working time for continuous rotary motion to reciprocating motion. This may be due to the use of thermally treated WaveOne Gold files, while the ProTaper Universal Retreatment system is manufactured from conventional NiTi alloy. Therefore, the flexibility of WaveOne Gold files caused them to bend instead of penetrating the filling material, resulting in increased working time. In contrast, the OTR motion did not affect working time when compared to continuous rotary motion due to the use of the ProTaper Universal Retreatment system in both motions. The reason may be attributed to the fact that the clockwise component of the OTR files' reciprocating motion aligns with the design of the ProTaper Universal Retreatment files, which also operate in a clockwise direction. Moreover, there might be a slight increase in the time required for retreatment when using OTR files due to the reciprocating motion, but this difference was not statistically significant. These results align with the findings of Jorgensen et al. in a previous study [25]. The results of this study differed from previous studies that indicated the superiority of reciprocating motion concerning working time during retreatment [9]. This difference may be attributed to variations in file alloy composition and cross-sectional design. Additionally, the results of this study differed from those obtained by Faus-Matoses et al., who reported no statistically significant differences when comparing the time required for retreatment using reciprocating motion or continuous rotary motion [18]. This difference may be due to the use of Reciproc blue files with a size of 40, where the larger diameter of the file reduces its flexibility, enhancing its penetration of the filling material. It is recommended to conduct similar studies on teeth with more complex anatomical structures, such as molars, which may present greater challenges in the context of endodontic retreatment. Additionally, it is suggested to perform similar studies on patients, assessing patient satisfaction with the time required for endodontic retreatment and evaluating additional clinical factors that may influence the endodontic retreatment duration.

Limitations

The primary limitation of this study lies in its focus on a single variable during the retreatment process, which is the time required. While this type of study could have targeted other aspects, such as the amount of apically extruded debris or the residual sealer remaining on the canal walls, this study concentrated solely on the time taken to evaluate the efficacy of the motion patterns mentioned.

## Conclusions

The use of OTR motion did not affect the time required for endodontic retreatment, as there were no statistically significant differences in preparation time when comparing the use of Protaper Universal Retreatment files with rotary motion versus OTR motion. Canal retreatment takes longer when using WaveOne Gold Primary files compared to instruments with continuous rotation and OTR motion.
